# Supplementary material for: A scoping review of the landscape of health-related open datasets in Latin America
Source: PLOS Digit Health. 2023 Oct 25;2(10):e0000368. doi: 10.1371/journal.pdig.0000368 (PMC10599518; doi:10.1371/journal.pdig.0000368)
Supplement: S1 File — (DOCX) [file pdig.0000368.s002.docx]

# S1 File:

## Question

What is the landscape of health-related open datasets in Latin America?

## Objective

In this scoping review we want to explore the current state of data sharing in Latin America, focusing on Latin America and health-related datasets. Specifically, in this review we aim to:

- Identify the existing open health datasets in Latin America through a mapping of the existing literature in Latin American countries.
- Identify the modalities, techniques, platforms and formats being used to share data in Latin America
- Highlight the initiatives and practices around the publication of open data in Latin America.
- Identify limitations and gaps around the current landscape of health data sharing in Latin America.
- Provide recommendations and suggestions to promote the use of open data in Latin America.

## Databases and Queries

### Scopus:

- Search terms and keywords:
  - ( TITLE-ABS-KEY ( "dataset" ) OR TITLE-ABS-KEY ( "data set" ) OR TITLE-ABS-KEY ( "database" ) ) AND ( TITLE-ABS-KEY ( "health*" ) ) AND ( TITLE-ABS-KEY ( "publicly available" ) OR TITLE-ABS-KEY ( "free of charge" ) OR TITLE-ABS-KEY ( "freely accessible" ) OR TITLE-ABS-KEY ( "publicly accessible" ) OR TITLE-ABS-KEY ( "open" ) ) AND ( TITLE-ABS-KEY ( "South America" ) OR TITLE-ABS-KEY ( "Latin America" ) OR TITLE-ABS-KEY ( "Brazil*" ) OR TITLE-ABS-KEY ( "Argentin*" ) OR TITLE-ABS-KEY ( "Colombia*" ) OR TITLE-ABS-KEY ( "Chile*" ) OR TITLE-ABS-KEY ( "Paraguay*" ) OR TITLE-ABS-KEY ( "Uruguay*" ) OR TITLE-ABS-KEY ( "Venezuela*" ) OR TITLE-ABS-KEY ( "Peru*" ) OR TITLE-ABS-KEY ( "Bolivia*" ) OR TITLE-ABS-KEY ( "Ecuador*" ) OR TITLE-ABS-KEY ( "Suriname*" ) OR TITLE-ABS-KEY ( "Guyan*" ) OR TITLE-ABS-KEY ( "Cuba*" ) OR TITLE-ABS-KEY ( "Dominican Republic*" ) OR TITLE-ABS-KEY ( "Costa Rica*" ) OR TITLE-ABS-KEY ( "El Salvador*" ) OR TITLE-ABS-KEY ( "Guatemala*" ) OR TITLE-ABS-KEY ( "Haiti*" ) OR TITLE-ABS-KEY ( "Honduras*" ) OR TITLE-ABS-KEY ( "Mexic*" ) OR TITLE-ABS-KEY ( "Nicaragua*" ) OR TITLE-ABS-KEY ( "Panama*" ) OR TITLE-ABS-KEY ( "Puerto Ric*" ) ) AND ( LIMIT-TO ( DOCTYPE , "ar" ) OR LIMIT-TO ( DOCTYPE , "cp" ) OR LIMIT-TO ( DOCTYPE , "dp" ) )
  - Result: Jun 21, 2023: 346 Documents

### Web Of Science

- Search terms and keywords:
  - (TS=("dataset" OR "data set" OR "database")) AND (TS=("health*")) AND (TS=("publicly available" OR "free of charge" OR "freely accessible" OR "publicly accessible" OR "open")) AND (TS=("South America" OR "Latin America" OR "Brazil*" OR "Argentin*" OR "Colombia*" OR "Chile*" OR "Paraguay*" OR "Uruguay*" OR "Venezuela*" OR "Peru*" OR "Bolivia*" OR "Ecuador*" OR "Suriname*" OR "Guyan*" OR "Cuba*" OR "Dominican Republic*" OR "Costa Rica*" OR "El Salvador*" OR "Guatemala*" OR "Haiti*" OR "Honduras*" OR "Mexic*" OR "Nicaragua*" OR "Panama*" OR "Puerto Ric*")) AND DT=(Data Paper OR Article OR Proceedings Paper)
  - Result: Jun 21, 2023: 214 Documents

### Pubmed:

- Search terms and keywords:
  - ((("dataset" [tiab]) OR ("data set"[tiab]) OR ("database" [tiab])) AND (("publicly available" OR "free of charge" OR "freely accessible" OR "publicly accessible")) AND (("South America" [tiab] OR "Latin America" [tiab] OR "Brazil*"[tiab] OR "Argentin*"[tiab] OR "Colombia*" [tiab] OR "Chile*" [tiab] OR "Paraguay*" [tiab] OR "Uruguay*"[tiab] OR "Venezuela*" [tiab] OR "Peru*" [tiab] OR "Bolivia*"[tiab] OR "Ecuador*"[tiab] OR "Guyana*"[tiab] OR "Suriname*"[tiab] OR "Cuba*"[tiab] OR "Dominican Republic*"[tiab] OR "Costa Rica*"[tiab] OR "El Salvador*"[tiab] OR "Guatemala*"[tiab] OR "Haiti*"[tiab] OR "Honduras*"[tiab] OR "Mexic*"[tiab] OR "Nicaragua*"[tiab] OR "Panama*"[tiab] OR "Puerto Ric*"[tiab])) ) AND ("Journal Article"[Publication Type] OR "Congress"[Publication Type] OR "Dataset"[Publication Type])
  - Results Jun 21, 2023: 140 results

## Inclusion Criteria:

- Studies published in academic journals, conference proceedings, and reputable sources.
- Studies that focus on health-related open datasets in Latin America.
- Studies that provide information on the availability, accessibility, and use of open health datasets.
- Studies that discuss the modalities, techniques, platforms, and formats used for sharing data in Latin America.
- Studies that highlight initiatives and practices related to the publication of open data in Latin America.
- Studies that identify limitations, gaps, and challenges in the current landscape of health data sharing in Latin America.

## Exclusion Criteria:

- Non-academic sources such as blog posts, opinion pieces, and news articles.
- Studies not focused on health-related datasets or not specific to Latin America.
- Studies that do not discuss the availability, accessibility, or use of open datasets.
- Studies that are not related to the modalities, techniques, platforms, or formats used for sharing data in Latin America.
- Studies that do not address initiatives and practices related to the publication of open data in Latin America.
- Studies that do not identify limitations, gaps, and challenges in the current landscape of health data sharing in Latin America.
