# Supplementary material for: A scoping review of the landscape of health-related open datasets in Latin America
Source: PLOS Digit Health. 2023 Oct 25;2(10):e0000368. doi: 10.1371/journal.pdig.0000368 (PMC10599518; doi:10.1371/journal.pdig.0000368)
Supplement: Attachment — Submitted filename: Peer_Review_PLOS_Digital_Health-Simon-Provost.pdf [file pdig.0000368.s003.pdf]

Peer Review

Open Data in Health Research: A Review of Latin American Datasets

Simon Provost, sgp28@kent.ac.uk
Ph.D. Student at the University of Kent, United-Kingdom

1 Research Overview and Initial Impressions

In the context of Latin America, the article conducts a comprehensive analysis of (Scopus, Web of Science, and PubMed) articles employing open health data. This analysis illuminates the current state of open health data in the region, focusing on its accessibility, utilisation, and overall quality. Out of 700 original research papers, the researchers identified a total of 141 filtered articles during the review process. As a result, the reader is assured that, at a minimum, a substantial amount of data has been examined in order to potentially conduct a fair analysis. In general, I viewed this as a positive aspect.

Therefore, the authors provide suggestions for enhancing the use of open data in Latin America. These recommendations include the cultivation of multidisciplinary collaboration and the promotion of a culture that encourages data sharing. Consequently, multiple metrics were covered while investigating these papers and datasets, allowing any member of the Latin American research community to begin with a fundamental understanding of these datasets and a solid foundation. Accordingly, I believe that the recommendations are clear and in the right direction.

The paper concludes with significant findings about the current status of open health data in Latin America, highlighting the need for improved infrastructure and cultural support to promote the open data movement.

Overall, I appreciated the paper because it was direct and to the point. As stated, movement in the proposed direction is without a doubt essential and urgent. Despite the fact that I believe the paper should be accepted, I would like the authors to consider the following suggestions.

2 Strengths, Weaknesses, and Overall Recommendations

The strengths of the paper are its comprehensive approach to analysing the current state of open health data in Latin America and its actionable recommendations. Additionally, the paper is well-organised and written clearly, facilitating comprehension of the research findings and their implications.

I do believe, however, that the urgent need to establish a regional benchmark baseline with the current data should also be emphasised. Given the need for data, if too many arrive but no one has benchmarked them to establish a baseline, new researchers will be required to start from scratch, and research will not be accelerated. This is elaborated upon in section 3.

In addition, Table 1 would have benefited from a direct link per dataset to a list of GitHub or GitLab projects that make active use of those data. Because, as a researcher interested in working with such data, if I select one from your table 1 and choose to conduct additional experiments on the given dataset, this could be a significant step towards accelerating the research on these open health data from Latin America if I have access to all the open-source repositories that have already worked with those data. Nonetheless, despite the fact that this could be a substantial rework, i.e. returning all 141 papers and reworking table 1 to include a cell-link list based on the new information. I will not consider this point as a primary significant change; therefore, you may consider this suggestion as you deem appropriate (e.g., in future work).

After the authors addressing the highlighted major recommendation below, I strongly endorse the publication of this paper.

3 Major Considerations and Proposed Recommendations

As stated previously, the paper provides a comprehensive analysis and recommendation regarding open health data in Latin America. However, it lacks a crucial component: a recommendation for a regional baseline and benchmarks for open health data in Latin America. Such benchmarks could enable the Latin American research community to follow a baseline while creating new algorithms or datasets to include in the baseline benchmarks and expanding the region's global open health data database. Due to this omission, new researchers may have to start from scratch, which could slow the rate of research. Moreover, these open-source benchmarks could have a significant impact on the Latin American research community, fostering collaboration and innovation as recommended elsewhere in the paper.

To increase the impact of the paper in this direction, we suggest adding the following between the recommendations titled "Improve Data Quality and Standardization" and "Foster a Culture of Data Sharing," i.e. between lines 225 and 226. However, the authors should be aware that what follows is merely one option that we have proposed based on our experience, and they are free to accept or reject it in favour of an alternative:

Automation is an essential component of research methodologies in today's data-driven environment. Therefore, we propose that Automated machine learning (Auto-ML) [1, 2] be recommended as a potent classification tool to deal with the vast amount of data proposed in the paper, or any other such tool that can enable automation and, if used correctly, can generate automatically whenever a new dataset is created and appended. The Auto-ML system could be healthcare-specific or a generic, state-of-the-art Auto-ML system, depending on the nature and characteristics of the data. We are withholding a specific system's recommendation because we have not thoroughly examined the data.

Second, such an application of Auto-ML recommendations to classify the proposed data can produce benchmark results as a second component. The research community in Latin America can then utilise this benchmark as a regional standard. Lastly, automating the benchmark can also be extremely beneficial. This could be hosted on open source platforms such as GitHub or Gitlab, enabling the community to collaborate directly with the regional benchmark: (1) add a new dataset, and, (2) based on a software engineering process, have the chosen Auto-ML system automatically train and output a baseline result on the dataset to be, (3) added to the regional-based benchmark automatically.

By implementing these recommendations, it would be possible to accelerate the development of research-based algorithms and encourage the generation of additional data, as all the proposed ones would be utilised and exhausted by the community faster.

Given our expertise in healthcare applications employing machine learning and Auto-ML techniques, we are confident that this recommendation is valuable and could contribute to the rapid expansion of the Latin American research community in regards to open health data.

References

[1] J. Waring, C. Lindvall, and R. Umeton, "Automated machine learning: Review of the state-of-the-art and opportunities for healthcare," *Artificial intelligence in medicine*, vol. 104, p. 101822, 2020.

[2] M.-A. Zöller and M. F. Huber, "Benchmark and survey of automated machine learning frameworks," *Journal of artificial intelligence research*, vol. 70, pp. 409–472, 2021.
