# Supplementary material for: A scoping review of the landscape of health-related open datasets in Latin America
Source: PLOS Digit Health. 2023 Oct 25;2(10):e0000368. doi: 10.1371/journal.pdig.0000368 (PMC10599518; doi:10.1371/journal.pdig.0000368)
Supplement: Attachment — Submitted filename: Response to Reviewers.pdf [file pdig.0000368.s004.pdf]

David Restrepo
Laboratory for Computational Physiology, Harvard-MIT Division of Health Science and Technology,
Massachusetts Institute of Technology,
Email: davidres@mit.edu
August 22, 2023

Dr. Crina Grosan
Academic Editor
PLOS Digital Health

Dear Dr. Grosan,

Re: Manuscript ID PDIG-D-23-00259
Title: "A Scoping Review of The Landscape of Health-Related Open Datasets in Latin America"

I hope this message finds you well. I would like to express my sincere gratitude to you and the reviewers for your valuable feedback and constructive comments on our manuscript entitled "A Scoping Review of The Landscape of Health-Related Open Datasets in Latin America," submitted to PLOS Digital Health. We appreciate the time and effort put into the review process, and we have carefully considered all the suggestions and criticisms provided.

We are pleased to submit a revised version of our manuscript in response to the reviewers' comments. Below, we address each of the points raised by the reviewers:

Journal Requirements

The changes are highlighted in blue in the “Revised Manuscript with Track Changes” file.

1. Reference List: We have reviewed the reference list to ensure its completeness and correctness.

2. Competing Interests: We have updated the 'Competing Interests' statement as per your recommendation. It now reads:

"I have read the journal's policy and the authors of this manuscript have the following competing interests: Leo Anthony Celi is the Editor-in Chief of PLOS Digital Health."

3. Funding Information: We have revised the Funding Information section to accurately reflect the funding received for this research.

4. Copyrighted Material: We have made the necessary changes to the figures and map as per your instructions. Figure 2 now includes a direct link to the base layer of the map and information regarding its terms of use and license.

Reviewer #1:

We would like to acknowledge the constructive criticism made by the reviewer. In response to the highlighted major recommendation, we have made significant additions to our manuscript.

The changes are highlighted in green in the “Revised Manuscript with Track Changes” file.

We recognize the critical importance of establishing a regional baseline and benchmarks for open health data in Latin America. To address this, we have included a new paragraph emphasizing the significance of benchmarks in our revised manuscript in the second paragraph of the section recommended by the reviewer (Improve Data Quality and Standardization):

"Incorporating benchmarks is extremely important in the context of open health data in Latin America. To facilitate this, we propose the use of open-source and free tools like GitHub or GitLab, together with programming languages like Python or R, to run benchmark models and ensure reproducibility. Benchmarks not only provide a standardized framework for evaluating the quality and performance of open health datasets but also serve as a foundation for ongoing research and collaboration. By establishing benchmarks, the Latin American research community can accelerate the development of research-based algorithms and encourage the generation of additional data, thus fostering innovation and collaboration."

We believe that this addition aligns well with the reviewer's concern about the need for benchmarks and emphasizes their role in promoting collaboration, innovation, and research acceleration. We propose the use of open source tools such as Python or R since although tools such as Auto-ML can speed up tasks, they are also an expense that not many institutions and researchers in Latin America can afford.

Reviewer #2:

The changes are highlighted in yellow in the “Revised Manuscript with Track Changes” file.

1. Limitation of Article Searches:

We recognize this limitation as one of the great weaknesses of our research in the last paragraph of the discussion:

"This review has limitations. The data sources for extracting the articles were limited to 3 databases (Scopus, Web Of Science, and PubMed), and despite the combined scope, some papers and databases may have been omitted, although we argue that databases should be “findable” if they are to be useful. It should also be taken into account that many of the data sources found do not come from scientific publications, but are data sources published by governments and/or local or international organizations."

To make this statement clearer, we added at the end of the paragraph the sentence:

"We understand that many of the open data sources in Latin America may not be present in scientific databases and therefore may have been excluded from this review. Other data sources such as open data repositories should be explored in future reviews."

2. Clarification of "Authors" Section: We have rephrased the section "Authors" and “Conclusions” to make it clear that we evaluated authors' affiliations rather than nationality.

We changed in “Authors” section:
“at least one Latin American author was included”
To:
“at least one author was working in a Latin American institution”

And in “Conclusion”:
“author’s nationality”
To:
“country of authors' affiliations”

3. Findable Datasets in Data Repositories: We have added a note about the possibility of datasets being findable only in data repositories and not as publications, as per your suggestion. This comment was addressed in the first point of the response to this review “1. Limitation of Article Searches”

4. Support Funding and Infrastructure: We have incorporated your recommendation about mentioning the role of funding agencies in mandating open data sharing. We added in the “Support Funding and Infrastructure” section the paragraph:

“While data sharing policies vary across the region, some countries are aligning with global practices by incorporating the obligation to open data release, often tied to funding support. These policies promote transparency and accessibility, highlighting the need for dedicated funding mechanisms and enhanced data infrastructure. These policies can foster data-sharing cultures and empower Latin America in the open data aspects.”

5. Rigorous De-identification: We added the next sentence at the end of the first paragraph of the “Improve Data Quality and Standardization” section:

“Practices, such as controlled access can also be used in the cases where de-identification of medical data is not possible. In certain cases, using credentialed data access, rather than fully open data, may be a suitable approach, ensuring that data security and ethical considerations are addressed.”

6. Risks of Data Sharing: We changed the paragraph in section “Foster data sharing culture” from:

“Encouraging a culture of data sharing is crucial for the advancement of open data initiatives in Latin America. Key steps to achieve this include recognition, academic or otherwise, and incentives for those who share data openly, such as funding opportunities. Health institutions should be made aware that sharing data is important and represents a benefit and not a risk.”

To:

“Encouraging a culture of data sharing is crucial for the advancement of open data initiatives in Latin America. Key steps to achieve this include recognition, academic or otherwise, and incentives for those who share data openly, such as funding opportunities. Health institutions should be made aware that sharing data is an important but delicate process. Incorporating data treatment policies to avoid risks related with data privacy and identification is vital in each institution. However, sharing data represents more benefits than risks for health institutions.”

In addition to the specific points mentioned above, we have carefully reviewed and revised the entire manuscript for clarity, grammar, and language.

We believe that these revisions significantly strengthen the quality and comprehensiveness of our manuscript. We are confident that the revised version now meets the publication criteria of PLOS Digital Health.

Thank you once again for the opportunity to revise and resubmit our manuscript. We look forward to your feedback on the revised version.

Thank you for your continued consideration.

Sincerely,

David Restrepo
Laboratory for Computational Physiology, Harvard-MIT Division of Health Science and Technology,
Massachusetts Institute of Technology
Telematics Department, University of Cauca
Email: davidres@mit.edu
